# Editorial: Iron and Neurodegeneration

**DOI:** 10.3389/fnins.2019.01382

**Published:** 2019-12-20

**Authors:** Giorgio Biasiotto, Massimiliano Filosto, Isabella Zanella

**Affiliations:** ^1^Department of Molecular and Translational Medicine, University of Brescia, Brescia, Italy; ^2^Section of Cytogenetics and Molecular Genetics, Clinical Chemistry Laboratory, ASST Spedali Civili di Brescia, Brescia, Italy; ^3^Neurology Unit, Centre for Neuromuscular Diseases and Neuropathies, ASST Spedali Civili di Brescia, Brescia, Italy

**Keywords:** iron, neurodegeneration, autophagy, neuroinflammation, exosomes

As a co-factor of many proteins, iron is involved in essential biological processes, from DNA synthesis to mitochondrial respiration, and oxygen transport. In the central nervous system (CNS), it is required for further crucial functions like myelination and synthesis of neurotransmitters. Iron can cycle between two redox states, Fe^2+^ and Fe^3+^. While this cycling is essential for crucial ox-redox reactions, it can be harmful, generating reactive oxygen species (ROS) through the Fenton reaction. Both brain iron deficiency and overload are noxious and in health conditions brain iron homeostasis is tightly regulated at the blood brain barrier (BBB) and in all cells. Brain iron increases in healthy aging, due to BBB dysfunctions or chronic inflammation that are typical of the elderly, but it raises also in several neurodegenerative diseases, in which iron dyshomeostasis and redistribution are frequently observed. Since distinct neurodegenerative disorders have their own etiology, pathogenesis and course, iron dysregulation often affects different cells, and pathways. However, common hallmarks may be identified as drivers or consequences of brain iron homeostasis disruption, like protein aggregation, impaired autophagy, ox-stress, ferroptosis, neuroinflammation, and altered immunity.

The aim of this Topic Issue was to provide an updated overview on the role of brain iron dysregulation under different neurodegenerative conditions. Several researchers contributed interesting point of views on this subject that continues to be a matter of debate among neuroscientists.

The central theme of the Topic is masterly illustrated by Ndayisaba et al., who provided an accurate picture of brain iron metabolism in both health conditions and aging. The authors reviewed shared dysfunctional pathways coexisting with iron accumulation or dyshomeostasis in different neurodegenerative disorders. They focused on dysfunctions of mitochondria, site of the synthesis of heme and Fe-S clusters (ISCs); protein misfolding and aggregation in which iron seems implicated; disruption of autophagy, possibly resulting in dysfunctional degradation of ferritin (FT), the main intracellular iron storage protein, and inadequate or excessive release of bioavailable iron; iron activation of microglia and astroglia and neuroinflammation; ferroptosis induction linked to iron dysregulation. The authors provided evidence for disease modification by modulators of iron homeostasis and for the usefulness of Magnetic Resonance Imaging (MRI) technology to study iron as early or disease progression biomarker.

The dual role of iron dyshomeostasis and protein aggregation neurotoxic effects in neurodegenerative disorders was thoroughly reviewed by Joppe et al., who suggested their mutual interplay in amplifying their detrimental effects. They provided evidence of an iron role in protein aggregation through its direct binding to amyloidogenic proteins like α-synuclein in Parkinson's disease (PD), Aβ and tau in Alzheimer's disease (AD), superoxide dismutase 1 (SOD1) and TAR DNA binding protein (TDP43) in Amyotrophic Lateral Sclerosis (ALS) and prion protein (PrP) in prion diseases. Authors highlighted the indirect iron role in the regulation of α-synuclein aggregation through ox-stress induction and autophagy inactivation and the modulation of secretase activity in amyloid precursor protein (APP) processing, Aβ release, and tau phosphorylation. They emphasized the impact of mutated SOD1 on iron dyshomeostasis and the influence of PrP on cellular iron deficiency and neuroinflammation. Finally, they reviewed updated clinical applications of iron chelators as symptomatic and neuroprotective agents in PD, AD, and ALS.

The connection between iron levels and the unfolded protein response (UPR) was explored in a research report (Healy et al.). The authors took advantage of their established neonatal hippocampal slice-based model for their study. They exposed slices to iron overload and found that iron loading induced UPR-related transcripts and loss of oligodendrocytes. These effects were partially prevented by UPR activation. *In silico* analysis of putative transcription factor binding sites within promoter regions of iron-related genes identified binding sites for UPR-associated transcription factors, suggesting further studies in this direction.

The role of ferroptosis was illustrated by further contributions. De Gregorio-Rocasolano et al. described iron transport to the brain through the BBB and iron uptake by brain cells, well-summarizing the actually known cellular receptors and the main proteins involved in intracellular iron handling in different brain cells. The authors then focused their attention on the role of iron in ischemic stroke (IS) and intracerebral hemorrhage (ICH), reviewing how iron can induce ROS production and ferroptosis and the beneficial effects of iron chelators and ferroptosis inhibitors in reducing neuronal death.

Wu et al. after depicting a detailed review of known molecular mechanisms involved in ferroptosis, showed intriguing evidence of its potential role in peripartum asphyxia and intraventricular/periventricular hemorrhage in preterm infants. The authors emphasized that the neonatal immature brain is more prone to ferroptosis, due to higher rate of oxygen consumption, higher concentration of polyunsaturated fatty acids and lower endogenous antioxidant defense mechanisms and proposed a new treatment approach, based on ferroptosis inhibition, encouraging future research in this unexplored direction.

Quiles del Rey and Mancias illustrated the ferroptotic role of nuclear receptor co-activator 4 (NCOA4), involved in ferritinophagy. Although its physiological role has been mainly studied in erythropoiesis, several studies showed a link between ferroptosis and ferritinophagy. Currently there is no direct evidence for ferritinophagy dysregulation in neurodegeneration, however both in health aging and neurodegenerative conditions brain iron increases and autophagy decreases and several neurodegenerative disorders due to mutations in proteins involved in the autophagy machinery are associated with brain iron accumulation. The dual role of NCOA4 in both iron homeostasis and autophagy in neurodegeneration offers new insights for future research.

The role of crucial proteins other than NCOA4 but strictly related to brain iron dyshomeostasis and neurodegeneration were reviewed in further contributions. FT is a 24-mer formed by the assembly of two different subunits, heavy (FTH) and light (FTL) chains, with distinct functions: while FTH has ferroxidase activity, FTL promotes iron mineralization. Hereditary neuroferritinopathy (HF), reviewed by Muhoberac and Vidal, is an autosomal dominant movement disorder with brain iron accumulation, caused by FTL gene mutations. The authors reviewed *in vitro* and *in vivo* models of HF, with an emphasis on functional anomalies of the mutant FTL and detailing the role of FTL aggregation, ROS generation, iron accumulation, ferritinophagy inhibition, and ferroptosis in its pathogenesis.

Ingrassia et al. focused on the role of divalent metal transporter 1 (DMT1), a transmembrane ferrous iron transporter that takes up non-transferrin bound iron (NTBI) at the plasma membrane and iron internalized through the transferrin (Tf)/transferrin receptor 1 (TfR1) cycle at the endosome membrane. Together with Tf, NTBI is a physiologic form of iron within interstitial fluid and cerebrospinal fluid (CSF) and the main source of iron uptake for some brain cells. The authors extensively reviewed its complex expression regulation, resulting from alternative splicing, transcriptional and post-transcriptional control and summarized proofs of upregulation during aging and neurodegenerations, providing evidence of its role in their pathogenesis.

All cells maintain a proper cytosolic iron level through the post-trascriptional regulation of TfR1, DMT1, FT, and the iron exporter ferroportin 1 (FPN1), through the binding/unbinding of iron regulatory proteins 1 and 2 (IRP1 and IRP2) to the iron regulatory elements (IREs) of TfR1, DMT1, FT, and FPN1 transcripts in iron depletion/repletion conditions. IRP2 is degraded in iron-replete conditions through proteasome via the F-box and leucine rich repeat protein 5 (FBXL5), in turn regulated by iron. IRP2 stability is also sensitive to ROS, although H_2_O_2_ treatment seems to stabilize IRP2 in H1299 cells or it does not affect IRP2 stability in other cells (Cairo et al., 2002; Hausmann et al., 2011). Conversely, Jiao et al. showed that H_2_O_2_ treatment induces IRP2 degradation through FBXL5 and proteasome, leading to reduced cellular iron uptake in the SH-SY5Y neuroblastoma cells. These intriguing findings needed further studies.

Llorens et al. reviewed the role of frataxin (FTX) in Friedreich's ataxia (FRDA), an early-onset neurodegenerative disease that affects CNS, heart and pancreas and caused by loss of function homozygous FTX mutations. The authors provided an updated overview on FTX functions in mitochondria and the mechanisms through which its loss of function can lead to iron dyshomeostasis. The authors provided an update on how FTX deficiency and iron overload can lead to neurotoxicity in FRDA through ROS-dependent and independent mechanisms.

Ceruloplasmin (CP) is a ferroxidase that oxidates exported cellular Fe^2+^ to Fe^3+^ for serum Tf binding and transport in blood stream. When this activity is compromised, cells retain iron. Patients with defective CP show progressive neurodegeneration due to iron accumulation. Ryan et al. studied CP and iron metabolism in wild-type and CP null animal models of cerebral ischemia. After ischemia, mice CP expression significantly increases in wild-type mice, while iron-related proteins were partly differently modulated in the two models. These data showed that the absence of CP generated iron dyshomeostasis and ox-stress and worsen the recovery of function when cerebral ischemia occurred.

Aceruloplasminemia is a rare autosomal recessive disorder with adult onset. The clinical features include also neurological symptoms directly proportional to brain iron accumulation. Marchi et al. reviewed recent literature, highlighting the importance of recognizing early evidence of this pathology to prevent the heavy burden of clinical manifestations. The therapeutic approach to aceruloplasminemia is deepened by the review of Piperno and Alessio, who analyzed the use of iron chelators to attenuate systemic iron overload and described the poor ability of these molecules to counteract neurological symptoms and the side-effect of worsening anemia. The authors presented recent studies in animal models in which parenterally administered CP showed a reduction of systemic iron overload and a promising neuroprotective effect.

The role of iron in neuroinflammation was depicted with intriguing points of view in some contributions.

Hepcidin is the master regulator of systemic iron metabolism. Hepcidin seems implicated on iron overload and neuroinflammation. It is expressed in the brain and where perhaps there is a contribution of the hepatic form when BBB leakage occurs like in inflammation. Hepcidin expression is low in brain cells, but increases in iron overload and inflammation. In this condition, hepcidin reduces brain microvascular endothelial cells ability to transport iron. Inflammation increases neuronal iron uptake and this condition is worsened by FPN1 degradation induced by hepcidin. The therapeutic potential of hepcidin needs additional studies since it could be of importance in the treatment of neurodegenerative diseases (Vela).

Healthy individuals maintain a balance between trained and tolerized immunity. Iron changes this balance during microbe infections due to the competition between host and pathogens. The strategy of innate immune cells to seize and accumulate iron in order to limit microbe iron availability could damage mitochondria, increase ROS, and generate autoimmune inflammation. Iron and aging could be responsible for intestinal and brain microvessel permeability, causing the migration of intestinal microbes to the CNS with disruption of immune tolerization, particularly deleterious in AD. Modification of trained and tolerized immunity using exosomes and nanoparticules charged with iron chelators and/or other therapeutic molecules could be useful to treat AD (Sfera et al.).

The increase of gut permeability permits microbiota to migrate in other tissues and stay as dormant microbes. Pretorius et al. suggested iron dysregulation and reactivation of dormant microbes as the trigger of AD. Iron dyshomeostasis observed in AD might increase ox-stress, cause the reactivation of dormant microbes and increase the amyloid formation. Bacteria could contribute to amyloid deposition and neuroinflammation. Glial cell activation and the increase of BBB permeability could fuel the inflammatory progression of AD.

Siotto et al. contributed with an interesting research article, showing a connection between peripheral inflammation, ox-stress, and iron homeostasis in patients with relapsing-remitting multiple sclerosis (RR-MS) and low disability. Analyzing ox-stress and iron metabolism through the measure of several biomarkers in peripheral blood, the authors found that in the early phases of the disease the ox-stress status was impaired and that disease-modifying therapy could affect iron homeostasis and ox-stress. Ashraf et al. studied CSF levels of melanotransferrin (MTf), an iron-binding protein belonging to the Tf superfamily that exists as both a plasmamembrane-anchored protein and a secreted form. Although its functions are currently unclear, it was found highly expressed in reactive microglia within senile plaques and significantly increased in the serum and CSF of AD patients. The authors hypothesized the association of baseline levels of CSF MTf to classic AD CSF biomarkers in a clinically well-defined cohort of AD patients, subjects with mild cognitive impairment (MCI) both converted and not converted to AD and cognitively normal subjects and found that MCI subjects that converted to AD had lower baseline MTf levels compared to non-converters and lower CSF MTf levels were associated with greater cognitive deficits and lower hippocampal volumes in AD converter, suggesting that iron homeostasis perturbations may be an early contribution to the disease process prior to acquiring AD.

The role of iron in AD pathogenesis was extensively reviewed by Liu et al. The authors described how iron can participate in the deposition of Aβ plaques through its effect on APP expression and cleavage, binding to Aβ, and accelerating its aggregation. Iron is also implicated in the deposition of tau tangles, another pathological feature of AD, since it binds tau and induces its phosphorylation and aggregation. Authors reviewed the potential of iron-related biomarkers for AD diagnosis and progression and classic and new therapeutic approaches based on the use of iron chelators.

The role of iron in PD pathogenesis was extensively analyzed in further contributions. MRI biomarkers are increasingly applied in the diagnosis of movement disorders. Neuromelanin-sensitive magnetic resonance imaging (NM-MRI) combined with nigrosome-1 (N1) imaging by quantitative susceptibility mapping (QSM) in the substantia nigra (SN) may be used to distinguish *de novo* PD from patients affected by essential tremor (ET) (Jin et al.). This study indicated that PD patients have greater SN iron content when compared to ET patients. Implications of iron dyshomeostasis in PD and Parkinsonisms were highlighted by Lee and Lee. Atypical parkinsonian syndromes are characterized by brain iron accumulation. Multiple system atrophy (MSA), progressive supranuclear palsy (PSP) and PD patients can be differentiated using MRI, analyzing iron-related signal changes in tomographic patterns of widespread iron deposition in deep nuclei. The authors described the reasons that can influence region specificity and underlined the pivotal importance of considering age-related, sub-structural and structure-specific patterns when MRI is used to investigate iron related neurodegeneration.

Another contribution analyzed PD patients, differentiated on the basis of symptomatology. The interesting study by Lian et al. improved our knowledge on iron-related proteins, serum and CSF inflammatory factors in PD. The authors demonstrated that tremor dominant PD patients showed increased interleukine-6 (IL-6) and iron levels and decreased FTL in CSF and increased IL-6 and FT serum levels when compared with postural instability and gait difficulty-dominant PD patients and controls. The authors concluded that an interconnection exists between dysregulated iron metabolism and inflammation in tremor dominant patients.

Dopamine was studied, considering its possibility to generate aminochrome and ROS, specifically in neurons that are characterized by pathological iron accumulation (Sun et al.). Authors observed that post-mortem tissue of brain from PD patients showed acidosis. Studies in the pH range of 7.4–6.5 indicated that iron increased toxic quinones like aminochrome. This effect was amplified by Fe^2+^ when compared to Fe^3+^ and aminochrome accumulation was more elevated in brain with acidosis. In acidic environment, the elevated iron redox cycling enhanced the amount of ROS in the presence of dopamine.

Nitric oxide (NO) supports physiological metabolism in the brain, but it has been linked to neurodegeneration when produced at high levels. NO metabolism is strictly connected to iron homeostasis, it regulates IRPs influencing the expression of iron-related proteins and promotes the S-nitrosylation of these proteins modifying their functions. The interdependence between NO and iron homeostasis, the modification of iron metabolism resulted by change in NO concentration in PD and AD and the chelation therapy effects were reviewed by Liu et al. Some biological questions remain however open. The improvement of the techniques used in NO detection and evaluation of iron redox state could progress this field.

Recent findings evidenced an interconnection between iron and calcium homeostasis in neurons. Iron activates calcium signaling with downstream activation of kinases that influence synaptic plasticity. Ox-stress mediated by iron increase excessively activates calcium signaling that impairs mitochondrial function and dysregulates mitochondrial iron metabolism. This anomalous crosstalk between iron and calcium homeostasis leads to neuronal death. Núñez and Hidalgo reviewed recent literature in the field, showing that the dangerous deregulation involved excitotoxicity, lipid peroxidation and damage of important mechanisms of iron and calcium pathways, contributing to neurodegenerative disorders, like AD and PD.

The role of iron in ALS was explored and reviewed by Petillon et al. The authors summarized evidence of iron accumulation and dysregulation in brain and spinal cord both in patients and animal models. The authors reviewed also studies on hepcidin levels in ALS founding contrasting results. Next, the authors dealt with the questions regarding the diversity of models, methodologies, biomarkers and heterogeneity of findings, critically reviewing the literature in order to identify the limits of currently published studies and suggesting relevant parameters to include in future studies on iron metabolism in ALS patients.

Halon-Golabek et al. contributed an original work in which mechanisms involved in iron-derived ox-stress in skeletal muscle are discussed, mainly focusing on ALS. They summarized evidence showing iron dysregulation in the skeletal muscle of subjects with muscle atrophy, suggesting that it may activate the ubiquitin-proteasome and autophagy-lysosome systems, protein degradation, and muscle mass loss. They provided evidence of a link between impaired insulin signaling pathways and iron accumulation. Further, they described iron dysregulation in the skeletal muscle of ALS patients and animal models, showing that it may contribute to ROS formation and impair muscle endocrine function, with an impact on the brain. Finally, they showed evidence of the protective role of exercise training on insulin sensitivity, iron homeostasis and myokine secretion.

Chiabrando et al. reviewed the pathological role of heme in neurodegenerative disorders and discussed the potential mechanisms generating toxicity in the brain, highlighting the importance of understanding these mechanisms to design therapeutic approaches. Traumatic brain injury is a causal factor for neurodegenerative diseases. Iron overload can take place through hemorrhage or microhemorrages due to heme-bound iron or labile free iron. Daglas and Adlard reviewed the role of iron in this pathological condition highlighting the interconnections with neurodegenerative diseases. Therapeutic options aimed to attenuate neuropathology and neurodegeneration risks involve the use of iron chelators that can penetrate BBB and antioxidants agents.

Taken together, the papers collected in this Issue present the most recent knowledge and experimental evidence about the role of iron in neurodegenerative disorders and offer new perspective and interesting hypotheses on this topic.

## Author Contributions

All authors listed have made a substantial, direct and intellectual contribution to the work, and approved it for publication.

### Conflict of Interest

The authors declare that the research was conducted in the absence of any commercial or financial relationships that could be construed as a potential conflict of interest.
